# Age modifies the association between sex and the plasma inflammatory proteome in treated HIV

**DOI:** 10.1172/JCI196869

**Published:** 2025-12-09

**Authors:** Rebecca A. Abelman, Samuel R. Schnittman, Natalia Faraj Murad, Adam B. Olshen, Gabriele B. Beck-Engeser, Noah Aquino, Gabrielle C. Ambayec, Edward R. Cachay, Joseph J. Eron, Michael S. Saag, Robin M. Nance, Joseph A. Delaney, Stephanie A. Ruderman, Richard D. Moore, Kenneth H. Mayer, Jeffrey M. Jacobson, Heidi M. Crane, Peter W. Hunt, for the CFAR Network of Integrated Clinical Systems (CNICS) Group

**Affiliations:** 1 UCSF, San Francisco, California, USA.; 2 Amgen, Thousand Oaks, California, USA.; 3 Mayo Clinic, Scottsdale, Arizona, USA.; 4 University of North Carolina, Chapel Hill, North Carolina, USA.; 5 University of Alabama at Birmingham, Birmingham, Alabama, USA.; 6 University of Washington, Seattle, Washington, USA.; 7 Johns Hopkins University, Baltimore, Maryland, USA.; 8 The Fenway Institute, Boston, Massachusetts, USA.; 9 Harvard Medical School, Boston, Massachusetts, USA.; 10 Case Western Reserve University, Cleveland, Ohio, USA.; 11 CNICS is detailed in Supplemental Acknowledgments.

**Keywords:** AIDS/HIV, Inflammation, Proteomics

## Abstract

**BACKGROUND:**

Among antiretroviral therapy–suppressed (ART-suppressed) people with HIV (PWH), women have higher levels of some inflammatory markers than men, but the effect of sex on the inflammatory proteome, and whether age modifies these differences, remain unclear.

**METHODS:**

Plasma inflammatory protein levels were assessed in ART-suppressed PWH from the Center for AIDS Research Network of Integrated Clinical Systems. The relationship between sex and plasma proteins — including 22 interferon-α response pathway proteins — was assessed, adjusting for confounders and assessing interactions by age.

**RESULTS:**

Of 922 participants, 162 (18%) were female. The median age was 47, above which the majority of women had undetectable plasma anti-Müllerian hormone levels, a biomarker of ovarian reserve. Age modified the influence of sex on the inflammatory proteome. Older age (>47) was associated with greater increases among women than men in 194 proteins. Interferon-α response proteins were higher in men in those ≤ 47 but higher in women in those > 47 (interaction *P* < 0.001). Among the 131 proteins associated with mortality risk (*q* < 0.05), only 5 differed by sex among those ≤ 47, while 79 differed by sex in those > 47, with nearly all being higher in women. Women had decreased mortality than men ≤ 47 (*P* < 0.001) but had similar mortality > 47 (*P* = 0.84).

**CONCLUSION:**

The menopausal transition appears to increase systemic type I interferon responses and inflammation in women with HIV, which may contribute to a loss of female advantage in mortality.

**FUNDING:**

NIH National Heart, Lung, and Blood Institute; National Institute of Neurological Disorders and Stroke; National Institute of Allergy and Infectious Diseases.

## Abstract 2

In people with HIV, the impact of sex on the plasma inflammatory proteome is highly dependent on age, with women exhibiting more inflammation than men primarily at older ages.

## Graphical abstract

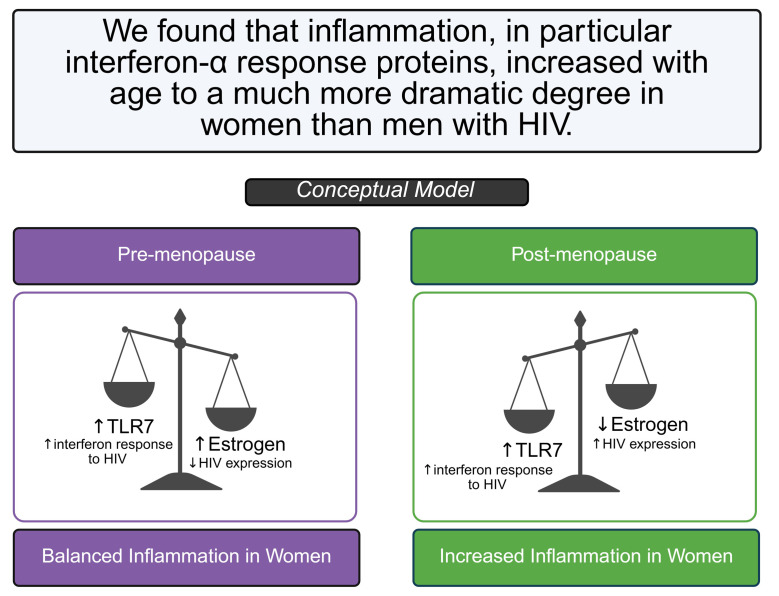

## Introduction

As people with HIV (PWH) age worldwide, the aging-related comorbidity burden for PWH has become an increasing health challenge; despite improvements in life expectancy, discrepant comorbidity-free years of survival remain lower than in people without HIV (1, 2). Furthermore, comorbidity burden is not distributed equally among PWH. Prior epidemiologic studies have found sex differences in PWH across age strata, with women with HIV demonstrating a higher comorbidity burden than men, particularly at older ages (3, 4). The life expectancy gap is also greater among women with HIV than men with HIV (5). The mechanisms underlying these sex- and age-associated differences are likely multifactorial, with immune activation playing a contributing role (6).

Several studies have demonstrated women with HIV have higher markers of immune activation and inflammation when compared with men with HIV, even when suppressed on antiretroviral therapy (ART) (7–9). One potential genetic mechanism for this difference lies in the greater expression among women of Toll-like receptor 7 (TLR7), which senses viral RNA in plasmacytoid dendritic cells (pDCs) and serves as a potent inducer of interferon response (10, 11). Prior studies have found that women had a stronger interferon response to HIV RNA stimulation of pDCs than men (10). This may be due to incomplete silencing of the X chromosome, where the TLR7 gene is encoded (11–14). Estrogen effects also appear to play a role in increased inflammation in women with HIV, with studies finding that estrogen promotes HIV latency (15) and that HIV expression from infected cells increases across the menopausal transition (16). Taken together, these findings suggest that sex assigned at birth and ovarian aging may play key roles in the immune response to HIV infection.

Prior work by our group in the Center for AIDS Research (CFAR) Network of Integrated Clinical Systems (CNICS) cohort assessed sex differences in 13 plasma markers of immune activation and inflammation and their association with the cardiovascular outcomes of acute myocardial infarction, venous thromboembolism, stroke, and death in ART-suppressed PWH. We observed that women had higher immune activation biomarkers than men and that these biomarkers appeared to predict clinical events more strongly in women than in men (17). Interestingly, age also played an important role, with women above the median cohort age of 47 displaying statistically significant increases in several immune activation biomarkers. These findings support the relationship between immune activation and clinical outcomes in PWH and that age may modify the impact of sex on inflammation. Whether there are important sex differences in the larger plasma inflammatory proteome in this setting, or relevant effect modifications by age, remains unclear.

To address this question, we leveraged a case-cohort study within the CNICS cohort. Using the Olink Inflammation Explore proximity extension assay (PEA) proteomics platform, we evaluated the effect of sex and its interaction with age on 363 unique plasma proteins in ART-suppressed PWH.

## Results

### Participant demographics and characteristics.

**Figure 1:**
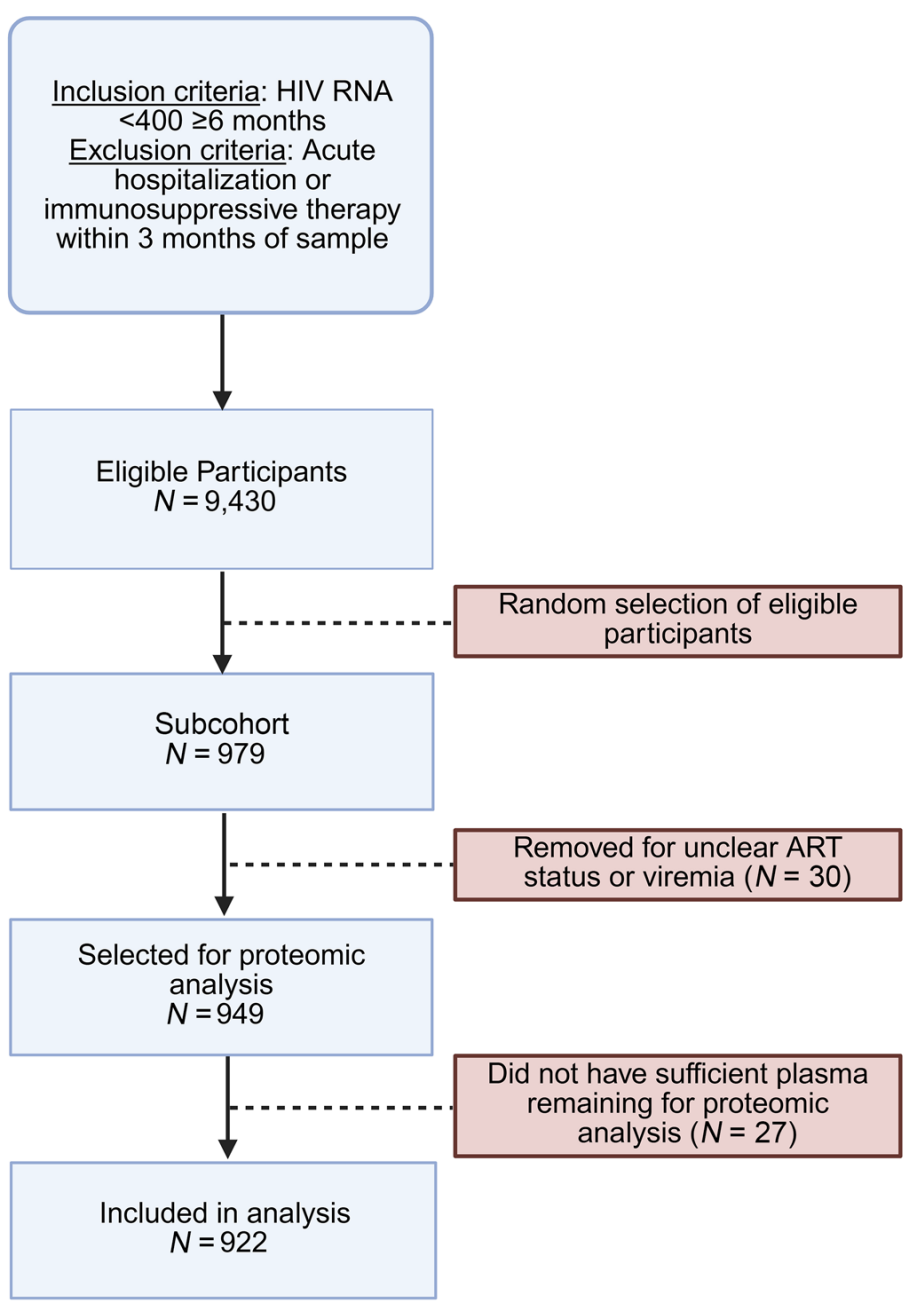
CONSORT diagram of included participants.

Of the 922 included participants in the subcohort (Figure 1), 162 (18%) were female sex at birth, and a plurality identified as Black (46%) (Table 1). The median age was 47 years (IQR 39, 53). Age did not differ by sex assigned at birth, but the racial breakdown differed by sex, with the majority of women identifying as Black (77%). Women also had a higher prevalence of obesity (52% versus 19%) and diabetes (19% versus 12%). As expected, women had a higher CD4^+^ T cell count than men (639 cells/mm^3^ versus 566 cells/mm^3^), but the nadir CD4^+^ T cell count did not differ by sex.

### Sex differences in the inflammatory proteome are modified by age.

**Figure 2:**
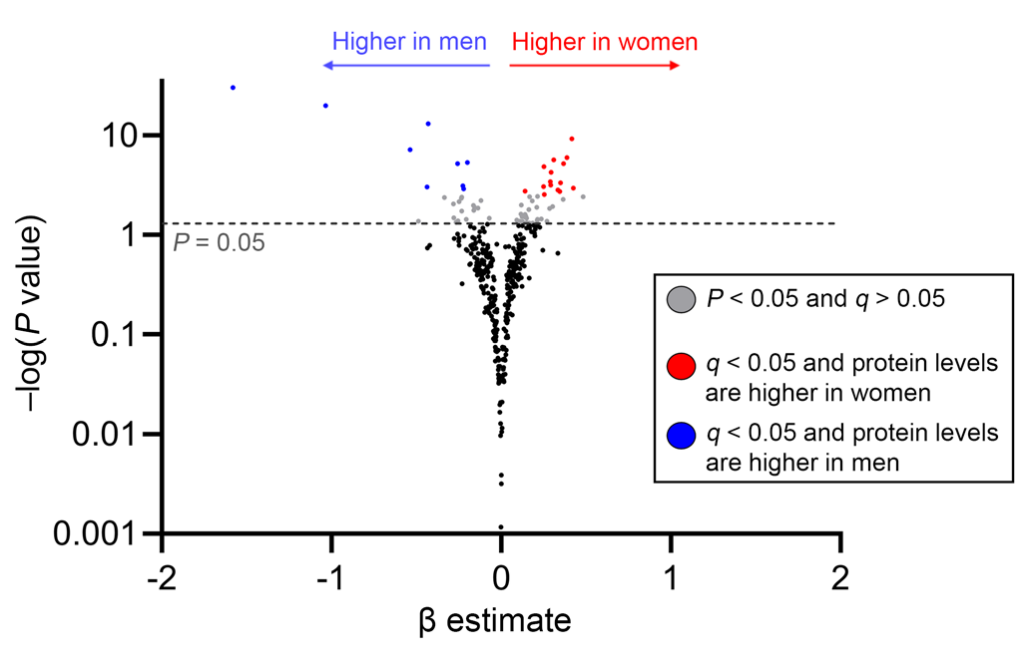
Plasma inflammatory proteome by sex at birth. Plasma levels of inflammatory and immunoregulatory proteins, assessed using the Olink Inflammation Explore panel, are compared between women and men after adjustment for CNICS site, age, race, nadir CD4^+^, men who have sex with men (MSM) status, smoking, IDU history, hepatitis C virus (HCV) history, obesity, and ASCVD risk score. Adjusted β coefficients (per 2-fold relative increase in protein levels) for women vs. men are plotted on the *x* axis and nominal significance level on the *y* axis, with proteins higher in women in red and those higher in men in blue.

**Figure 3:**
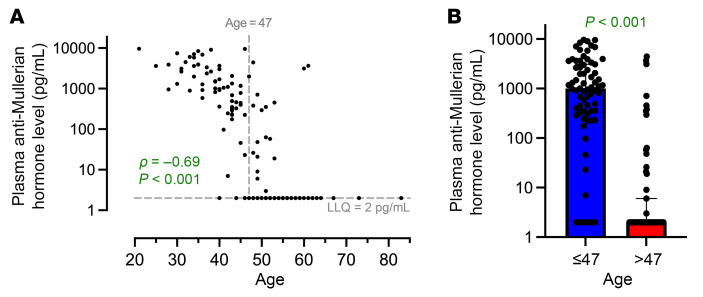
Plasma anti-Müllerian hormone levels by age in ART-suppressed women with HIV. (**A**) Plasma anti-Müllerian hormone (AMH) levels are plotted by age among 162 women in the subcohort, and correlation was assessed with a Spearman’s rho. The vertical dotted line denotes the median age of the cohort of 47. (**B**) Plasma AMH levels are plotted in those above and below the median age of 47, with the colored boxes denoting median value and error bars denoting the interquartile range (*P* for Wilcoxon’s rank-sum text).

**Figure 4:**
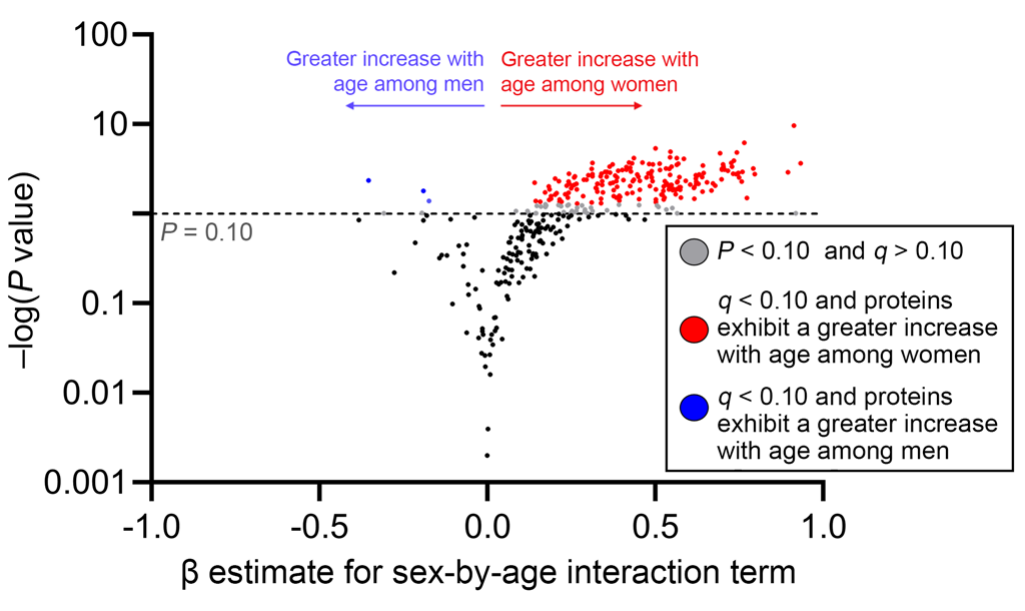
Plasma inflammatory proteome associations with age are modified by sex at birth. The degree to which sex at birth modified the association between age (≤47 vs. >47) and plasma inflammatory proteins was assessed with an interaction term, adjusting for CNICS site, race, nadir CD4^+^, MSM status, smoking, IDU history, HCV history, obesity, and ASCVD risk score. Adjusted β coefficients (per 2-fold relative increase in protein levels) for the sex-by-age interaction term are plotted on the *x* axis and nominal significance level on the *y* axis, with positive numbers (and red color) indicating a greater increase with age among women and negative numbers (and blue color) indicating a greater increase with age among men.

Without stratifying by age, after adjustment for potential confounders and false discovery rate (FDR) correction, we identified only 24 (7%) of the 363 proteins differed by sex (Figure 2). The proteins assessed with their respective β coefficients, *P* values, and *q* values are available in Supporting Data Values; supplemental material available online with this article; https://doi.org/10.1172/JCI196869DS1 Several inflammatory (e.g., IL-6) and immune activation proteins (e.g., CD70) were higher in women than men, while some immunoregulatory proteins (e.g., integrin β6 [ITGB6]) were lower in women. We next sought to determine whether there was evidence of sex-age interaction, dichotomizing age above and below the median age of 47. Importantly, the majority of women older than 47 had undetectable plasma AMH levels, suggesting late perimenopausal or menopausal status, while most women younger than 47 had detectable AMH levels (Figure 3). Using a cutoff of *q* < 0.10 for the sex-by-age interaction term, there was evidence of interaction in 194/363 (53%) proteins (Figure 4 and Supplemental Figure 1). The impact of older age on the inflammatory proteome was far stronger in women than men, with the vast majority of proteins increasing to a greater degree in women than men with older age. Additional adjustment for low-level viremia or restricting to those with an HIV RNA ≤ 40 copies/mL did not change the primary inferences regarding interaction between age and sex on the plasma proteome (Supporting Data Values). Gene ontology enrichment analysis revealed that pathways involved in both innate and adaptive immune activation tended to increase to a greater degree with age among women than men (Supplemental Figure 2).

**Figure 5:**
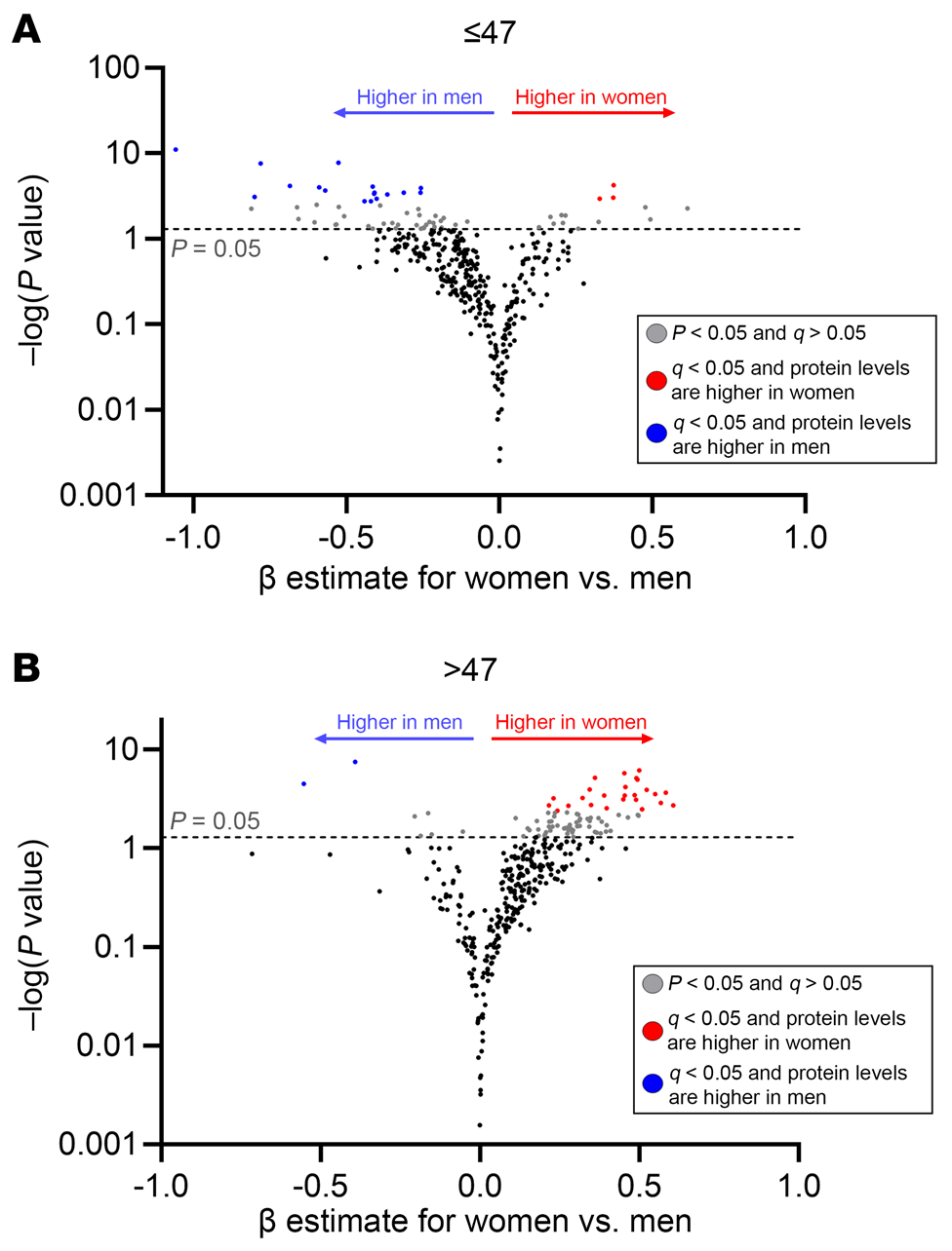
Sex differences in inflammatory proteome stratified by age. Plasma levels of inflammatory and immunoregulatory proteins are compared between women and men after adjustment for CNICS site, age, race, nadir CD4^+^, MSM status, smoking, IDU history, HCV history, obesity, and ASCVD risk score. Adjusted β coefficients (per 2-fold relative increase in protein levels) for women vs. men are plotted on the *x* axis and nominal significance level on the *y* axis, with proteins higher in women in red and those higher in men in blue among those younger (**A**) vs. older than age 47 (**B**).

Given this striking evidence for interaction between sex and age on the inflammatory proteome, we performed stratified analyses above and below the median age of 47. Among those younger than 47, more proteins were higher in men than in women at the *q* < 0.05 threshold (Figure 5A), many of which tended to be immunoregulatory, including ITGB6. In those above age 47, however, women tended to have higher levels of far more proteins than men at the FDR-corrected *q* < 0.05 level, of which the majority were pro-inflammatory (Figure 5B).

### Qualitative interaction between age and sex in the interferon-α pathway.

Since HIV expression from cells is known to increase in women as they cross the menopausal transition (16), and women are known to have a more robust type I interferon response to HIV than men (10, 14, 18), all 22 interferon-α–induced proteins in the panel were also specifically assessed. Strikingly, levels of most proteins in the Hallmark interferon-α response pathway increased to a greater degree with age in women than men.

**Figure 6:**
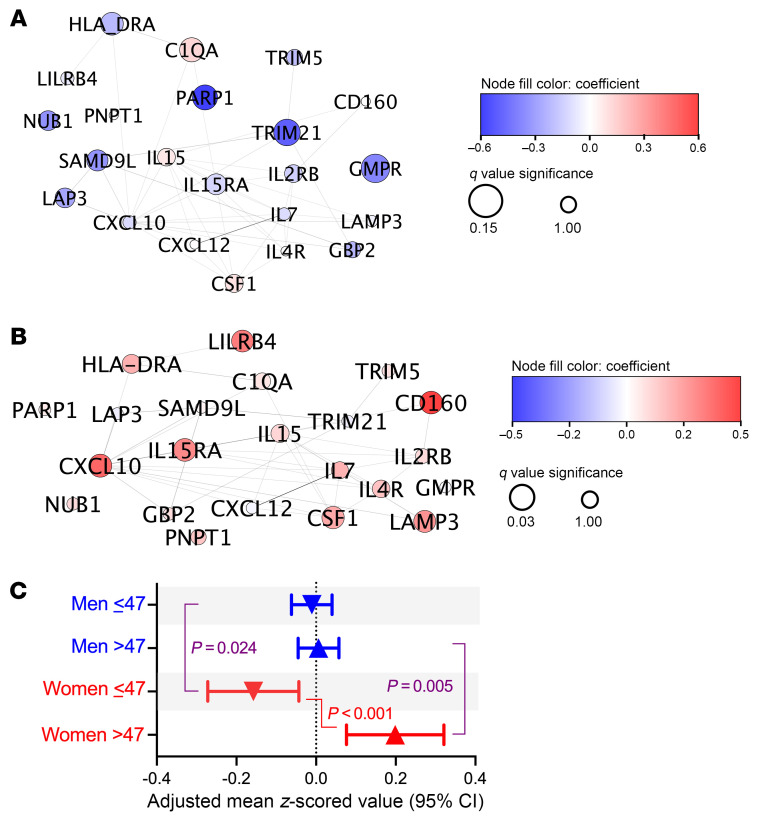
Qualitative interaction in the association between sex and age on interferon-α response protein levels. All 22 Hallmark interferon-α response pathway proteins represented in the Olink Inflammation Explore panel were *z*-scored and assessed for interaction by sex and age (≤47 vs. >47) after adjustment for CNICS site, race, nadir CD4^+^, MSM status, smoking, IDU history, HCV history, obesity, and ASCVD risk score. The β coefficients for female vs. male sex (red indicating a greater increase among women vs. blue for men) in the age ≤ 47 (**A**) and > 47 (**B**) strata are depicted in STRING protein network map for all 22 proteins in the pathway. A composite mean interferon-α response protein level score was calculated among all 22 *z*-scored proteins, and adjusted means were calculated for each age-sex stratum and compared, highlighting minimal changes with age among men but statistically significant changes with age among women (**C**).

In the stratified analysis, in those ≤47, the levels of most interferon-α–induced protein levels were higher in men (Figure 6A), while in those >47, nearly all proteins were higher in women (Figure 6B). To provide a quantitative assessment of interaction across all 22 proteins, we created an interferon-α response score as the mean of all *z*-scored proteins in the pathway and compared adjusted mean values across the age and sex strata (Figure 6C). While there was little evidence for a difference by age in the interferon-α response score in men, older age was associated with a dramatic increase in interferon-α score among women. Indeed, the interferon-α response score was higher in men in those ≤47 (*P* = 0.024) but higher in women in those >47 (*P* = 0.005, *P* for interaction < 0.001).

**Figure 7:**
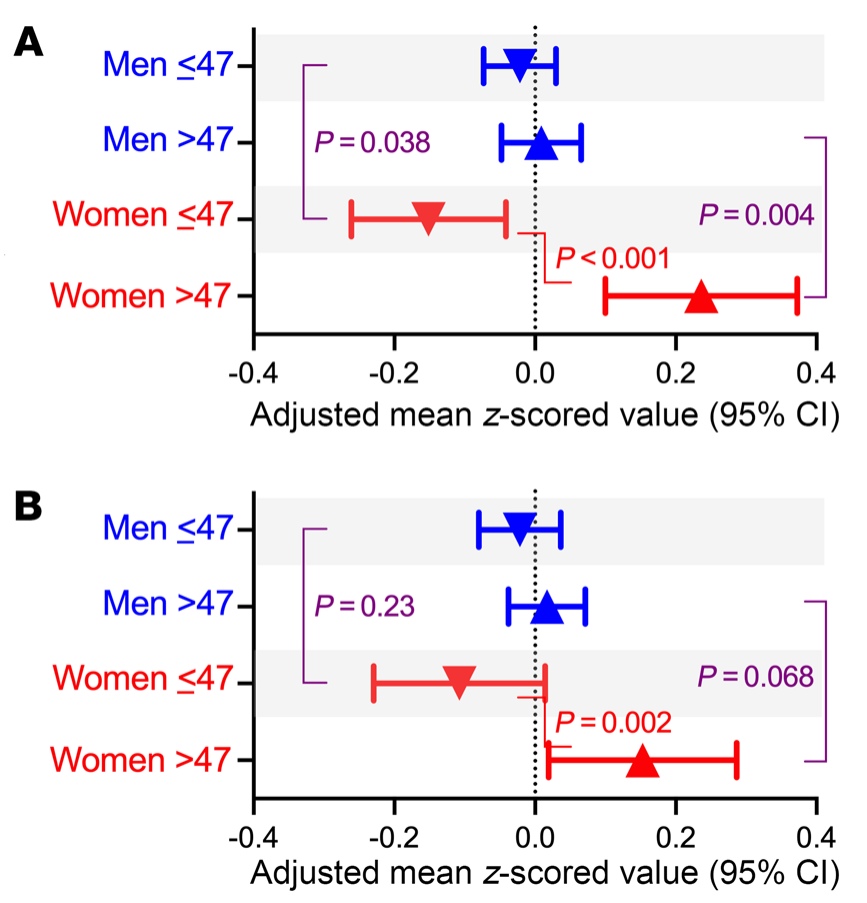
Qualitative interaction in the association between sex and age on IL-6 and coagulation pathway protein levels. Composite mean IL-6 and coagulation response protein level scores were calculated among all *z*-scored proteins, and adjusted means were calculated for each age-sex stratum and compared. For the IL-6 pathway, similar to the interferon-α pathway, there were minimal changes with age among men but statistically significant changes with age among women (**A**). There were similar findings in the coagulation pathway (**B**). Both models were adjusted for race, smoking status, IDU history, HCV serostatus, nadir CD4^+^, ASCVD risk score, MSM status, obesity, and CNICS site.

Given these findings, we created similar scores for Hallmark IL-6 and coagulation pathways. Both of these pathways had evidence of interaction by sex and age (Figure 7, A and B). Due to many of the proteins overlapping between these pathways, we found high correlation between the interferon-α response composite score with IL-6 and coagulation response scores (rho: 0.87, *P* < 0.0001 and rho: 0.83, *P* < 0.0001, respectively) and thus could not separate these pathways analytically.

### Clinical significance of age-sex interactions in inflammatory proteins.

**Figure 8:**
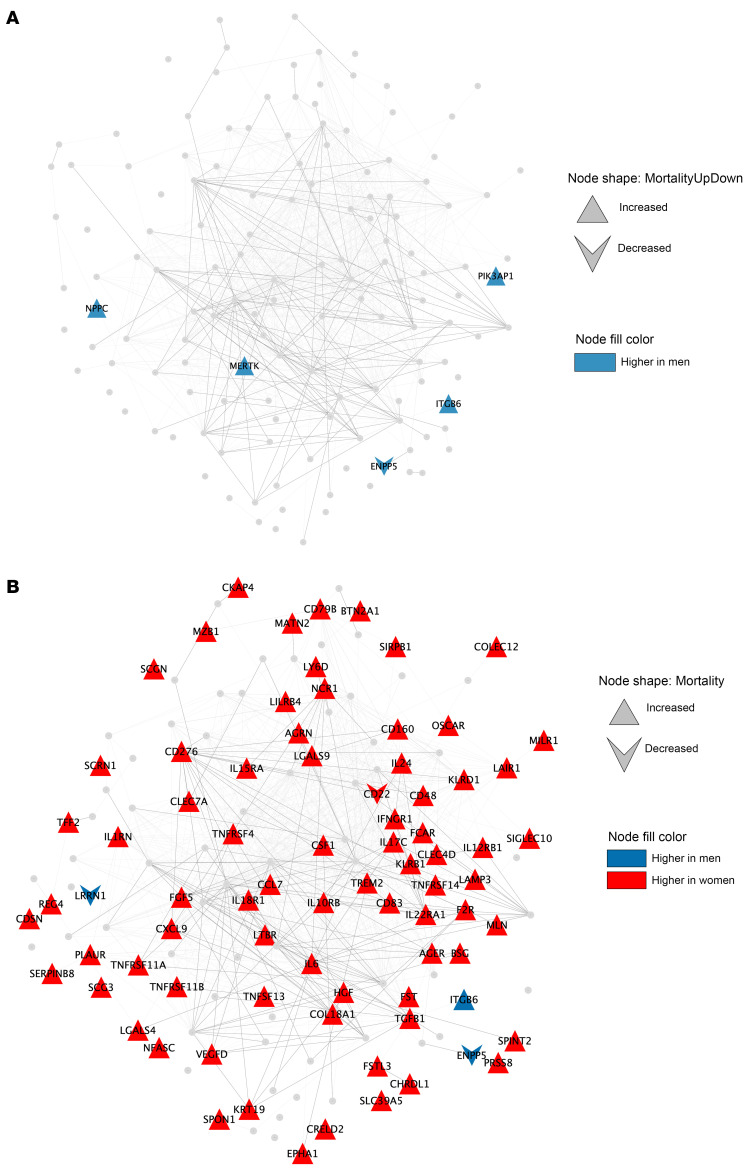
Sex differences in mortality-associated plasma proteins stratified by age. STRING protein network maps are shown for all 131 mortality-associated proteins in the panel, coloring each protein that was significantly different by sex at the FDR-corrected *q* < 0.05 level (blue higher in men, red higher in women) in those with age ≤ 47 (**A**) and in those with age > 47 (**B**). Proteins associated with increased mortality in the cohort are denoted with upward-pointing triangles, and proteins associated with decreased mortality are denoted by downward-pointing arrows.

**Figure 9:**
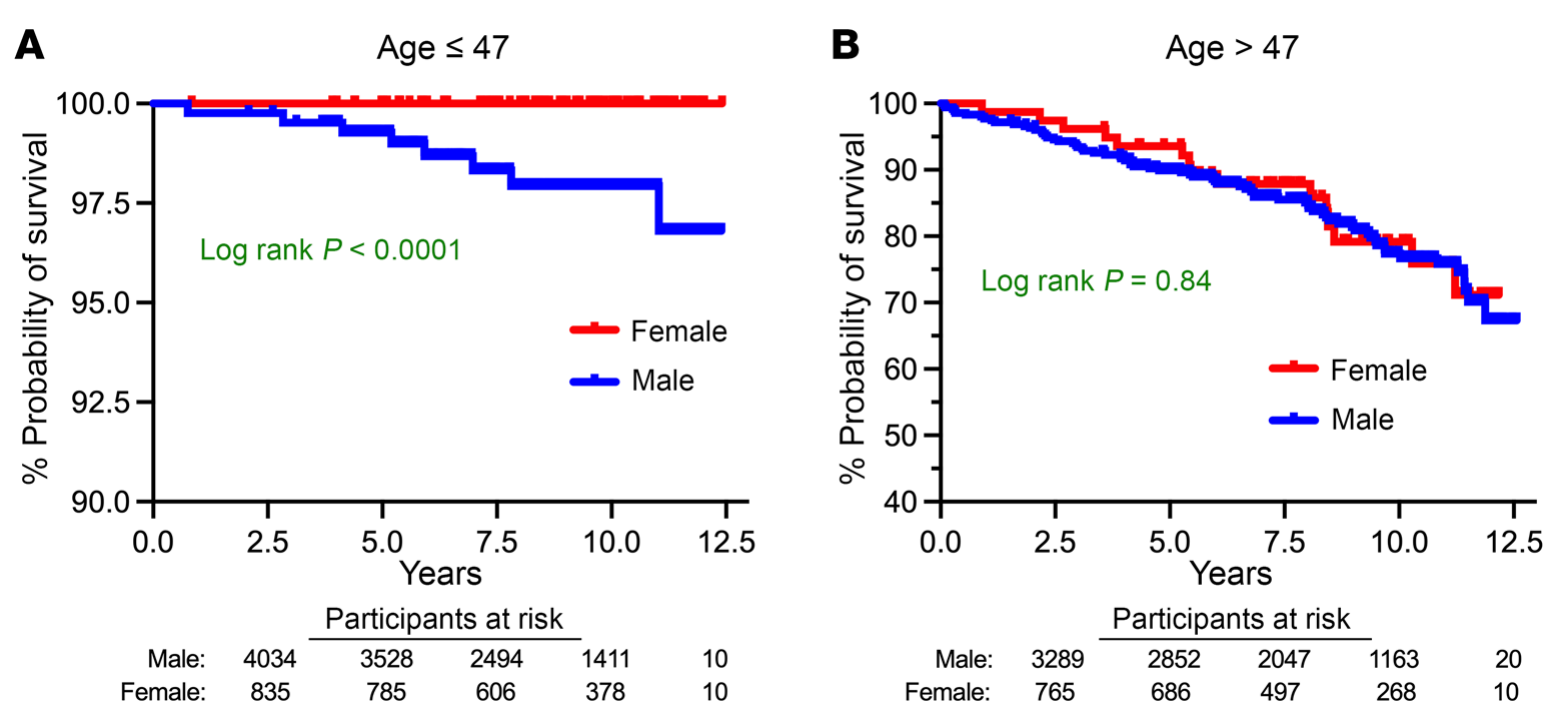
Sex-age interaction in mortality. Kaplan-Meier analysis plots of time to nonaccidental death, incorporating sample weights and testing differences by age with a log-rank test in participants below (**A**) and above the median age of 47 (**B**) are shown, with red denoting females and blue denoting males. In the table below each plot, the number of participants at risk using sampling weights is reported.

In the overall cohort of 922 participants, 103 died of a nonaccidental cause over a median 9 years of follow-up, of which 16 were female. In the mortality analysis, 127 (35%) of all proteins assessed were associated with increased hazard of mortality and 4 (1.1%) with decreased hazard of mortality after adjustment and FDR correction. When restricting the analysis to these 131 mortality-associated proteins, among those younger than 47, only 5 differed by sex, and all were higher in men (Figure 8A). Strikingly, among those older than 47, 79 (60%) differed by sex at the *q* < 0.05 level, with almost all of them higher in women. Furthermore, nearly all the proteins that were higher among women in the older age group were associated with increased mortality (Figure 8B). Only 3 proteins were higher in men in the older age stratum, and 2 of them (LRNN1 and ENPP5) were associated with decreased mortality. These findings were mirrored in Kaplan-Meier curves stratified by sex above and below the age of 47. In those under the age of 47, women had a lower mortality than men (*P* < 0.0001), whereas among those older than 47, women had a similar mortality as men (*P* = 0.84, Figure 9). Collectively, these data suggest that among older PWH, women have plasma proteomic profiles associated with greater mortality risk than men, which coincides with a loss of “female advantage” in mortality that is evident in younger participants.

### The relationship between inflammatory proteins and mortality risk is not statistically significantly modified by sex.

Since the higher levels of mortality-associated proteins in women than men in the older age stratum might have less clinical importance if the relationship between these proteins and mortality were weaker among women than men, we explored whether sex might modify the relationship between inflammatory proteins and mortality. We found no evidence that sex modified the association between any protein and mortality at the FDR-corrected *q* < 0.10 level. In fact, the interaction terms tended to favor stronger relationships between biomarkers and mortality in women than in men (Supplemental Figure 3A), including pathways involved in innate and adaptive immune activation (Supplemental Figure 3B).

## Discussion

We found that among ART-suppressed PWH, age dramatically modified the relationship between sex at birth and inflammation. While men tended to have more inflammation than women among younger PWH, among those older than 47, women tended to have much higher levels of inflammation than men. The interaction by age was particularly striking for interferon-α response proteins, which increased with age to a much more dramatic degree among women than men. Furthermore, putatively postmenopausal women with HIV had consistently higher levels of mortality-associated proteins than similarly aged men with HIV. These findings may suggest a mechanism to explain the loss of “female advantage” in life expectancy in PWH as well as a potential menopausal effect that may be responsible for exacerbating these sex differences.

Several studies have previously demonstrated sex differences in inflammatory biomarkers in PWH. Among PWH, women with HIV have higher immune activation biomarkers than men (7–9, 17), as well as a more robust type I interferon response to HIV, likely due to higher expression of the X-linked TLR7, which often escapes X inactivation in female cells (10, 12, 18, 19). Estrogen-related factors also appear to play a role, with evidence that estrogen may suppress HIV transcription (20). Indeed, Gianella et al. found that HIV transcription from infected cells increased in women with HIV across the menopausal transition, an effect that was not seen in similarly aged men (16). Collectively, these prior observations suggest that while women are predisposed to respond to HIV expression with a more robust type I interferon response, they have less HIV expression from cells during premenopause given the latency-promoting effects of estrogen. Conversely, as systemic estrogen levels decline with menopause, HIV expression from the reservoir increases, and since women are predisposed to respond with a more robust type I interferon response, type I interferon responses and inflammation would be expected to increase dramatically. That is what was observed here. We found that women tended to have lower inflammation than men at younger ages but consistently higher inflammation than men at older, putatively postmenopausal ages. We also found evidence of interaction by age and sex with IL-6 and coagulation pathways, which are biologically linked to the interferon pathway. Interferon-α may induce IL-6 production (21, 22) and markers of coagulation (23, 24), further corroborating our findings. We should note that not all prior studies of younger PWH have found lower type I interferon response proteins in putatively premenopausal women than men. For example, in a study of much younger PWH in sub-Saharan Africa, Streeck et al. found higher levels of CXCL10 in likely predominantly pre- or perimenopausal women (median age 39) than men (8). This may reflect differences in host genetics or other environmental exposures, including other prevalent infections that may tip the balance of type I interferon responses, overwhelming the protective effects of estrogen on suppressing HIV expression.

The sex differences in inflammatory proteins we observed at older ages are also likely to be clinically significant. When restricting to proteins that were associated with increased mortality, the vast majority were higher among women than men in the older age stratum. Higher levels of mortality-associated proteins in women might not have a clinical consequence if the relationship between these proteins and mortality were weaker in women than men, but that is not what was seen here. Indeed, while we found no statistical evidence that sex modified the association between inflammatory proteins and mortality, the relationships tended to be, if anything, stronger in women.

While most epidemiologic studies conducted in the general population find that women have a longer life expectancy than men (25), in several studies evaluating the survival trends in PWH, women have similar life expectancies to men and a greater “life expectancy gap” than men when compared with the general population (5, 26). No data currently exist suggesting that among PWH, postmenopausal women are at a greater mortality risk than men of a similar age. However, in our analysis, we found that women under the age of 47 demonstrated lower mortality than men whereas putatively postmenopausal women above the age of 47 had a similar mortality to men. Taken together, these findings support that a potential mechanism for the loss of “female advantage” in life expectancy among PWH may be through increased inflammation, perhaps mitigated by estrogen’s HIV latency–promoting effects at premenopausal ages and unmasked with menopause-associated estrogen depletion.

Prior studies have found a menopausal effect on immune activation in women with HIV. In a study evaluating the contribution of self-reported menopause to immune activation biomarkers in women with HIV, Peters et al. found that menopausal women with HIV had higher sCD14 and sCD163 levels, markers of monocyte and M2 macrophage activation, respectively, than their premenopausal counterparts (27). Prior studies in PWH have also found that sCD163 is an independent marker of all-cause mortality, with women with HIV demonstrating higher levels of sCD163 than men with HIV, and that sCD163 levels had a stronger relationship with mortality (28). In our analysis, the vast majority of women older than our median age of 47 had undetectable plasma AMH levels, consistent with late perimenopause or menopausal status, while most younger women had detectable levels. While a single AMH level without self-reported menstrual status data cannot be used to determine menopausal status, this suggests that the median age of the cohort coincided with entry into perimenopause. These findings suggest that menopausal status may have important effects on the plasma inflammatory proteome even when accounting for age, though this merits further study.

This study has several limitations. While we were able to see a strong sex effect, women represented only 18% of the cohort, which may have underpowered our ability to detect an association between sex and clinical outcomes. It is possible that residual confounding or unmeasured confounders also contributed to the findings seen here given the observational nature of our study, but we cannot think of an obvious alternative explanation for the interactions between age and sex on the inflammatory proteome observed here. While we had single measurements of AMH, we did not have serial measurements or self-reported menstrual status data to allow accurate determination of menopausal status. We also did not measure HIV expression in this cohort. Strengths of this analysis include the diverse nature of the cohort, which includes women and men with HIV across the United States. This study is distinct in its proteomic data on a large cohort of PWH with adjudicated clinical events and the inclusion of sex hormone data to assist with the determination of menopausal status, and highlights the needs of an understudied population, women with HIV.

The impact of sex on the plasma inflammatory proteome is highly dependent on age among ART-suppressed PWH, with women exhibiting more inflammation than men primarily at older ages. Whether menopause contributes to unmasking these sex differences requires further study and is of high clinical importance, as many of these pathways are associated with increased mortality. These findings might also suggest a protective role of estrogen replacement therapy among postmenopausal women with HIV, and a clinical trial to address this question is in development in the AIDS Clinical Trial Group (NCT06856174).

## Methods

### Sex as a biological variable.

As the objective of this study was to evaluate sex differences, sex was included as a biological variable. This study enrolled both cis-gender and transgender participants. Due to the small number of transgender participants resulting in low power, we only evaluated the cis-gender women in the analyses stratified by sex.

### Study design and population.

This study leveraged a case-cohort study within CNICS, a national cohort study integrating clinical data, laboratory data, and serial biospecimen collection from PWH across 8 CFAR sites (29). All enrolled adult PWH who had viral suppression from ART (HIV RNA < 400 copies/mL) for at least 6 months and had an available plasma aliquot after January 1, 2010, were eligible for inclusion. Study participants who had an acute infection, hospitalization, or receipt of immunomodulatory or immunosuppressive therapy within 3 months of plasma sampling were excluded.

From 9,430 eligible ART-suppressed participants, we randomly sampled 1,000 participants, of whom 922 had sufficient plasma remaining for analysis, comprising the subcohort. Participants in the subcohort were also followed for incident mortality, informed by the national death index.

Given the objective of the parent study to evaluate the association of inflammatory proteins with the long-term risk of clinical outcomes in ART-suppressed PWH, the first available plasma specimen after 6 months of ART-mediated viral suppression was selected as time zero for the study. For the mortality analysis, participants were followed from the date of plasma sampling to the date of censoring, i.e., the date of death or the last laboratory or clinic visit date. Accidental deaths were also censored.

### Proteomic measurements.

Inflammatory proteome measurements were obtained for 363 unique plasma proteins in singlicate on cryopreserved plasma using the Olink Inflammation Explore Panel (Olink), a PEA platform with high sensitivity and specificity (30). Relative protein levels are expressed in standardized Npx units for each protein, with each unit reflecting a 2-fold increase in protein level.

### AMH levels.

To evaluate whether age 47 could be used to approximate the age of menopause, AMH levels, a biomarker of ovarian reserve, were tested in cis-gender female participants. Plasma samples stored at –80°C were run in singlicate using the pico-AMH assay (Ansh Labs). Values were plotted by age to evaluate when AMH values became undetectable, signifying entry into perimenopause.

### Statistics.

The relationship between sex assigned at birth and plasma proteins was assessed with linear regression models adjusted for age, site, race, nadir CD4^+^, MSM status, smoking (ever smoker, yes/no), IDU history (ever IDU, yes/no), HCV history, obesity status (body mass index > 30 kg/m^2^), and ASCVD risk score. Covariates were selected a priori based on covariates that might plausibly be in the causal pathway between sex and inflammation among PWH. Given effect modification by age in our prior work in the cohort (17), age-sex interaction terms were generated, dichotomizing age above versus below the median age of 47, which also approximates the age of menopause for women with HIV (31).

To assess the clinical relevance of sex differences in inflammatory proteins, we restricted to proteins that predicted a shorter or longer time to nonaccidental death (i.e., censoring deaths known to be due to accidents or trauma) in Cox proportional hazards models adjusted for the VACS Index (32) and CNICS site at the FDR-corrected *q* < 0.05 level. To explore whether sex modified the relationship between inflammatory proteins and mortality, we used sex-by-protein interaction terms. Hazard ratios for the interaction term were reported as a direct measure of the degree to which sex modifies the association between biomarkers and mortality. To further evaluate whether age modified the impact of sex on mortality risk, we performed Kaplan-Meier analysis of time to nonaccidental death, incorporating sampling weights, testing differences by sex with a long-rank test, and stratifying by age.

Statistical tests (both *P* and *q* values) with an α level of 0.05 were considered significant for all analyses except the interaction terms, which used an α level of 0.10. All *P* values were adjusted for multiple comparisons by controlling the FDR using the Storey Q method (33). Known biologic relationships between statistically significant proteins (*q* < 0.05) were plotted using STRING network maps (https://string-db.org/) (34) with Cytoscape software (v3.10.3). Pathway enrichment analysis was performed using the C5: Gene Ontology sets from MSigDB (https://www.gsea-msigdb.org/gsea/msigdb), restricting to annotated pathways including the proteins represented in the Olink panel and further restricting to Biologic Process and immunologic pathways given the scope of our hypothesis (35). Given known sex differences in type I interferon pathways, as well as hypothesized changes in this pathway during menopause, all 22 Olink panel proteins in the Hallmark interferon-α pathway (M5911) were also assessed (10, 13), and a composite interferon-α response score was calculated as the mean of all *z*-scored Npx values. To assess additional pathways known to be induced by type I interferons, composite scores for Hallmark IL-6 response pathway proteins (*n* = 22) and coagulation pathway proteins (*n* = 7) were also assessed. Sex-by-age interactions were also explored for these composite variables as above. All data analyses were conducted using R (36).

### Study approval.

This CNICS study received approval from the University of California, San Francisco, Institutional Review Board as well as those boards from the participating sites. All study participants were informed about the study and provided written informed consent prior to study participation.

### Data availability.

The proteins assessed with their respective β coefficients, *P* values, and *q* values are available in Supporting Data Values. Additional supporting data are available in the BioStudies database (http://www.ebi.ac.uk/biostudies) under accession number S-BSST2240 (37).

## Author contributions

RAA helped develop the research question, interpreted the data, and wrote the manuscript. SRS helped with interpretation of the data and the writing of the manuscript. NM performed the statistical analysis and guided the interpretation of the results for the manuscript. ABO performed the statistical analysis and guided the interpretation of the results for the manuscript. GBBE was the full research specialist for PWH’s lab. GBBE helped with the Olink analysis for this project. NA also works in the lab with PWH. NA assisted with the Olink analysis for this project. GCA was a research specialist for PWH’s lab. GCA helped perform the Olink analysis for the project. ERC is a coinvestigator in the CNICS cohort; he contributed to the interpretation of data. JJE is a coinvestigator in the CNICS cohort; he contributed to the interpretation of data. MSS is a coinvestigator in the CNICS cohort; he contributed to the interpretation of data. RMN is a research specialist that works with the CNICS cohort. RMN provided the causes of death data and contributed to the interpretation of data. JAD contributed to the interpretation of data, particularly the causes of death data. SAR contributed to the interpretation of data. RDM is a coinvestigator in the CNICS cohort; he contributed to the interpretation of data. KHM is a coinvestigator in the CNICS cohort; he contributed to the interpretation of data. JMJ is a coinvestigator in the CNICS cohort; he contributed to the interpretation of data. HMC is a coinvestigator in the CNICS cohort; she contributed to the interpretation of data. PWH is a coinvestigator in the CNICS cohort. PWH secured funding for the study; oversaw the biomarker studies; assisted with refining the research question, data management, statistical analysis, and interpretation of the study results, in addition to providing input on the preparation of this manuscript.

## Funding support

This work is the result of NIH funding, in whole or in part, and is subject to the NIH Public Access Policy. Through acceptance of this federal funding, the NIH has been given a right to make the work publicly available in PubMed Central.

- NIH National Heart, Lung, and Blood Institute (NHLBI) (R01HL126538 and R01HL167658 to HMC, JAD, and PWH; R01HL180319 to PWH, HMC, JAD, and RAA; R56HL160457 to PWH; K12HL143961 to RAA).
- NIH National Institute of Neurological Disorders and Stroke (NINDS) (R01NS126086 to PWH).
- NIH National Institute of Allergy and Infectious Diseases (NIAID) (R24AI067039 to ERC, JJE, MSS, RDM, KHM, JMJ, HMC, and PWH; P30AI027763 to PWH; K24AI145806 to PWH).

## Supplementary Material

Supplemental data

ICMJE disclosure forms

Supporting data values

## Figures and Tables

**Table 1:**
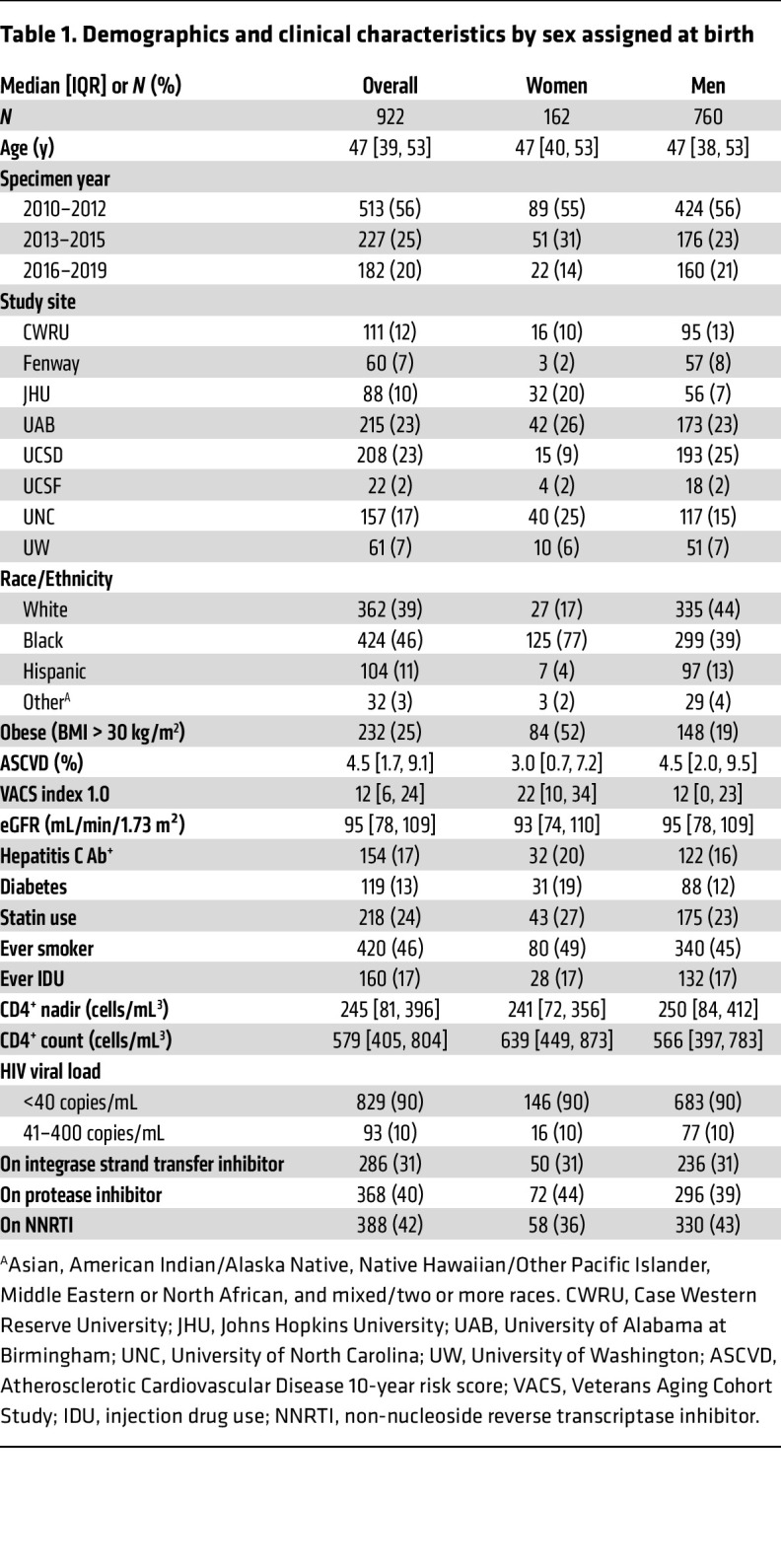
Demographics and clinical characteristics by sex assigned at birth

## Version 1 12/09/2025

In-Press Preview

## Version 2 02/02/2026

Electronic publication
