# Supplementary material for: Age modifies the association between sex and the plasma inflammatory proteome in treated HIV
Source: J Clin Invest. 2025 Dec 9;136(3):e196869. doi: 10.1172/JCI196869 (PMC12867131; doi:10.1172/JCI196869)
Supplement: Supplemental data [file jci-136-196869-s264.pdf]

A

B

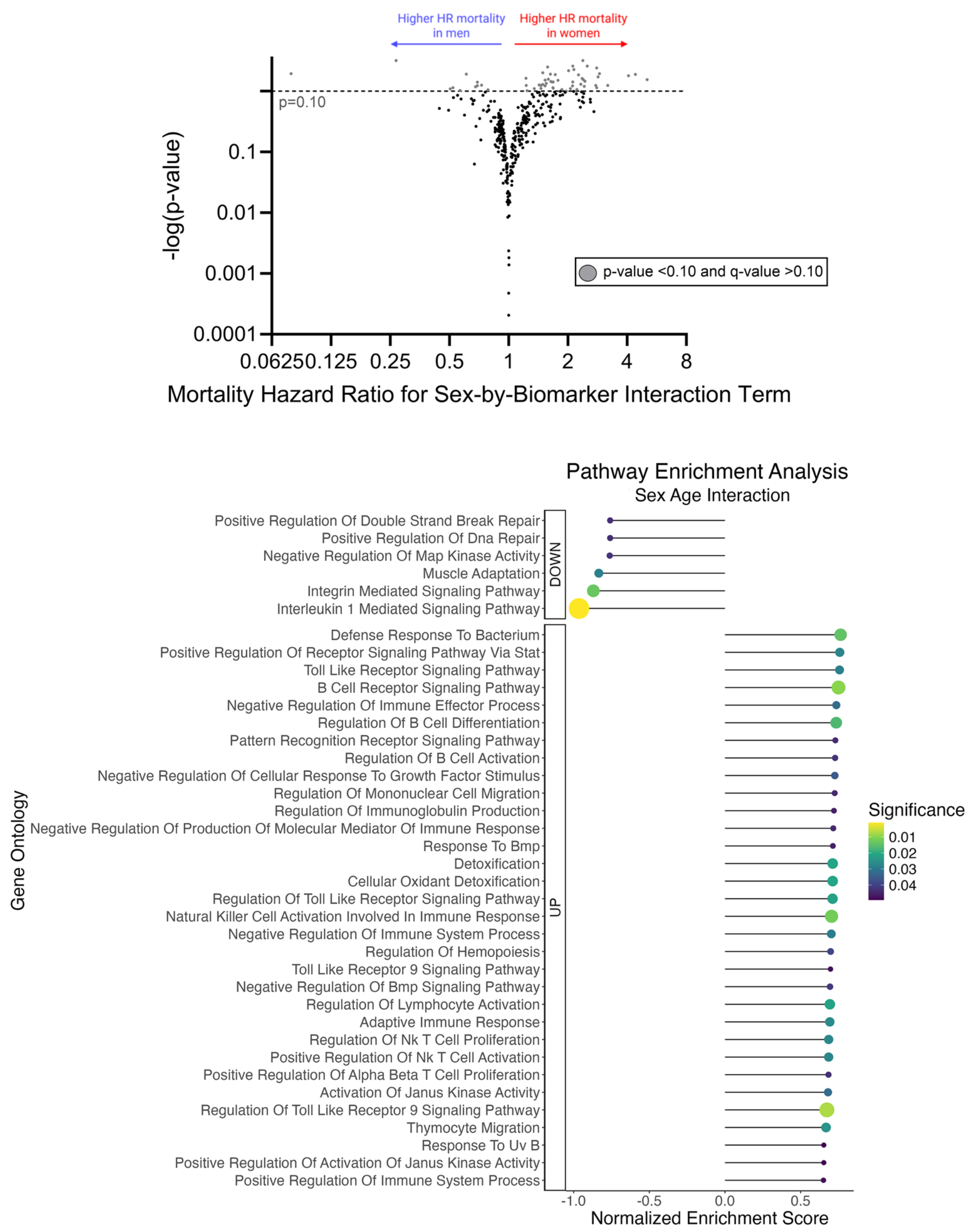

Higher HR mortality in men
Higher HR mortality in women
p=0.10
-log(p-value)
0.1
0.01
0.001
0.0001
0.0625
0.125
0.25
0.5
1
2
4
8
p-value <0.10 and q-value >0.10
Mortality Hazard Ratio for Sex-by-Biomarker Interaction Term
Pathway Enrichment Analysis
Sex Age Interaction
Gene Ontology
DOWN
Positive Regulation Of Double Strand Break Repair
Positive Regulation Of Dna Repair
Negative Regulation Of Map Kinase Activity
Muscle Adaptation
Integrin Mediated Signaling Pathway
Interleukin 1 Mediated Signaling Pathway
UP
Defense Response To Bacterium
Positive Regulation Of Receptor Signaling Pathway Via Stat
Toll Like Receptor Signaling Pathway
B Cell Receptor Signaling Pathway
Negative Regulation Of Immune Effector Process
Regulation Of B Cell Differentiation
Pattern Recognition Receptor Signaling Pathway
Regulation Of B Cell Activation
Negative Regulation Of Cellular Response To Growth Factor Stimulus
Regulation Of Mononuclear Cell Migration
Regulation Of Immunoglobulin Production
Negative Regulation Of Production Of Molecular Mediator Of Immune Response
Response To Bmp
Detoxification
Cellular Oxidant Detoxification
Regulation Of Toll Like Receptor Signaling Pathway
Natural Killer Cell Activation Involved In Immune Response
Negative Regulation Of Immune System Process
Regulation Of Hemopoiesis
Toll Like Receptor 9 Signaling Pathway
Negative Regulation Of Bmp Signaling Pathway
Regulation Of Lymphocyte Activation
Adaptive Immune Response
Regulation Of Nk T Cell Proliferation
Positive Regulation Of Nk T Cell Activation
Positive Regulation Of Alpha Beta T Cell Proliferation
Activation Of Janus Kinase Activity
Regulation Of Toll Like Receptor 9 Signaling Pathway
Thymocyte Migration
Response To Uv B
Positive Regulation Of Activation Of Janus Kinase Activity
Positive Regulation Of Immune System Process
-1.0
-0.5
0.0
0.5
Normalized Enrichment Score
Significance
0.01
0.02
0.03
0.04

Supplemental Figure 1: A STRING protein network map is shown for all proteins in the panel, coloring each protein for which the sex-by-age interaction term was significant at the FDR-corrected q<0.05 level according to its beta coefficient (red denoting greater increases with age among women and blue denoting greater increases with age among men).

Network Map for Protein-Age Associations Modified by Sex

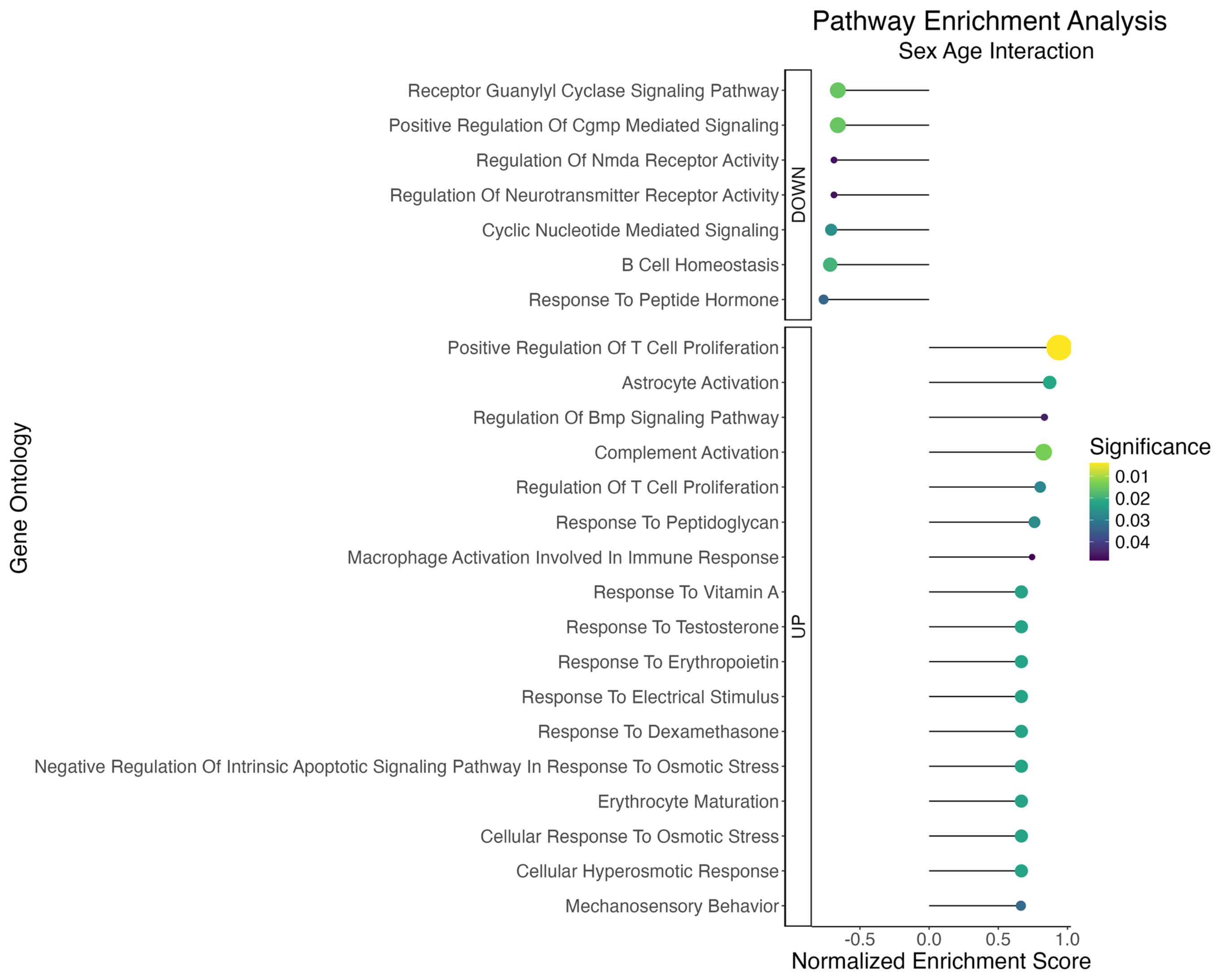
Pathway Enrichment Analysis
Sex Age Interaction
Gene Ontology
DOWN
Receptor Guanylyl Cyclase Signaling Pathway
Positive Regulation Of Cgmp Mediated Signaling
Regulation Of Nmda Receptor Activity
Regulation Of Neurotransmitter Receptor Activity
Cyclic Nucleotide Mediated Signaling
B Cell Homeostasis
Response To Peptide Hormone
UP
Positive Regulation Of T Cell Proliferation
Astrocyte Activation
Regulation Of Bmp Signaling Pathway
Complement Activation
Regulation Of T Cell Proliferation
Response To Peptidoglycan
Macrophage Activation Involved In Immune Response
Response To Vitamin A
Response To Testosterone
Response To Erythropoietin
Response To Electrical Stimulus
Response To Dexamethasone
Negative Regulation Of Intrinsic Apoptotic Signaling Pathway In Response To Osmotic Stress
Erythrocyte Maturation
Cellular Response To Osmotic Stress
Cellular Hyperosmotic Response
Mechanosensory Behavior
-0.5
0.0
0.5
1.0
Normalized Enrichment Score
Significance
0.01
0.02
0.03
0.04

Supplemental Figure 2: Pathways Changing to a Greater Degree with Age in Women. A gene ontology enrichment analysis (among all pathways containing proteins in the panel) was performed on the sex-by-age interaction term; pathways associated with greater increases with age among women with positive normalized enrichment scores and those with greater increases among men with negative enrichment scores; and nominal p values are depicted by the legend.

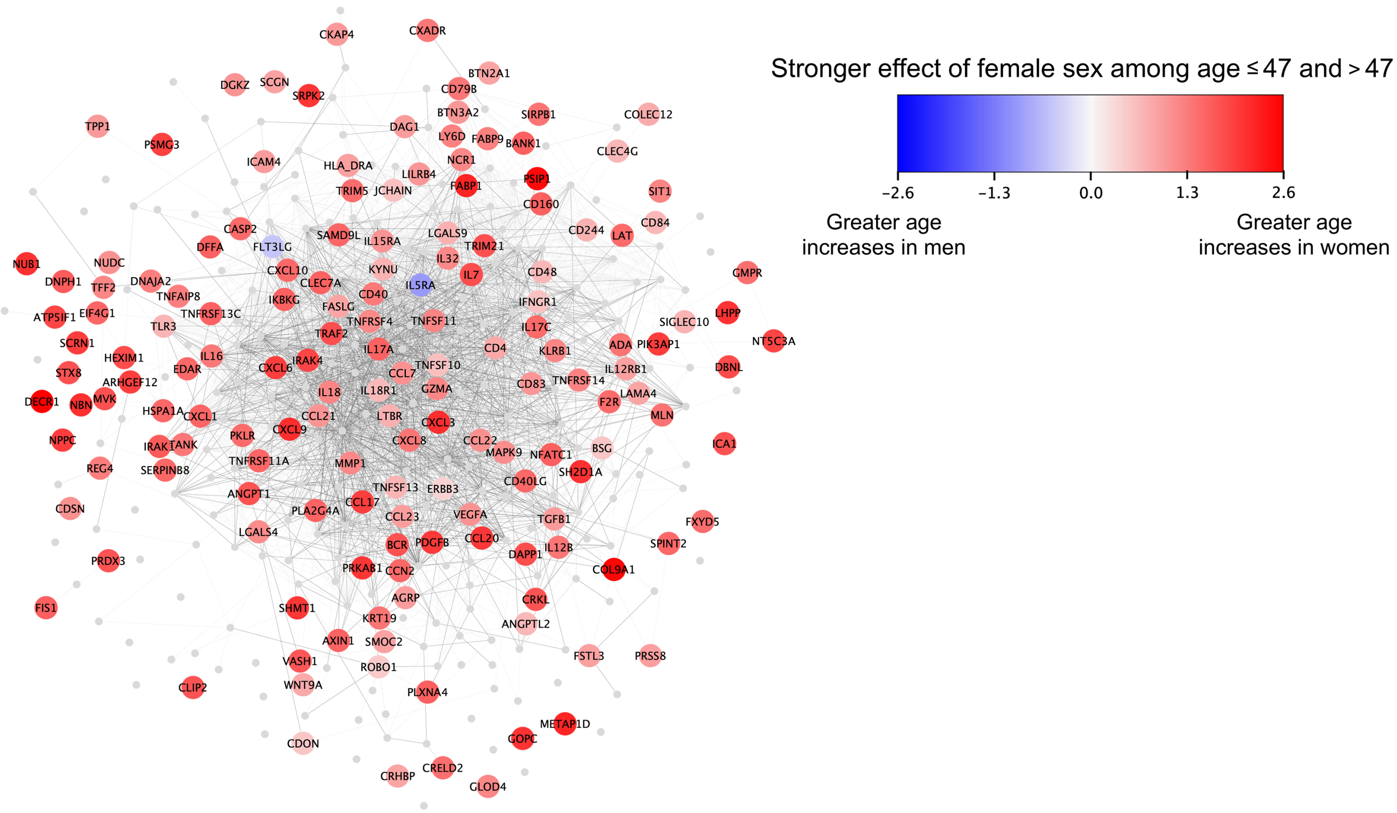

Stronger effect of female sex among age ≤ 47 and > 47
−2.6
−1.3
0.0
1.3
2.6
Greater age increases in men
Greater age increases in women
CKAP4
CXADR
BTN2A1
DGKZ
SCGN
SRPK2
CD79B
BTN3A2
SIRPB1
COLEC12
TPP1
DAG1
LY6D
FABP9
BANK1
PSMG3
CLEC4G
ICAM4
HLA_DRA
NCR1
LILRB4
PSIP1
TRIM5
JCHAIN
FABP1
SIT1
CD160
CASP2
CD244
CD84
SAMD9L
LGALS9
LAT
DFFA
FLT3LG
IL15RA
TRIM21
NUB1
NUDC
IL32
CXCL10
KYNU
CD48
GMPR
DNPH1
DNAJA2
IL7
TFF2
CLEC7A
IL5RA
TNFAIP8
IKBKG
CD40
IFNGR1
FASLG
EIF4G1
TNFRSF13C
LHPP
ATP5IF1
TLR3
TNFRSF4
TNFSF11
SIGLEC10
IL17C
TRAF2
NT5C3A
SCRN1
ADA
PIK3AP1
IL17A
CD4
KLRB1
HEXIM1
IL16
IRAK4
DBNL
EDAR
CXCL6
TNFSF10
STX8
IL12RB1
CCL7
ARHGEF12
TNFRSF14
CD83
GZMA
IL18
IL18R1
LAMA4
DECR1
MVK
NBN
F2R
HSPA1A
CXCL1
LTBR
MLN
CCL21
CXCL3
CXCL9
PKLR
CCL22
NPPC
CXCL8
ICA1
IRAK1
TANK
BSG
MAPK9
NFATC1
TNFRSF11A
MMP1
REG4
SERPINB8
SH2D1A
TNFSF13
CD40LG
ERBB3
ANGPT1
CCL17
CDSN
PLA2G4A
VEGFA
CCL23
TGFB1
FXYD5
LGALS4
CCL20
BCR
PDGFB
SPINT2
IL12B
DAPP1
PRDX3
PRKAB1
COL9A1
CCN2
AGRP
CRKL
FIS1
SHMT1
KRT19
ANGPTL2
AXIN1
SMOC2
FSTL3
PRSS8
VASH1
ROBO1
CLIP2
WNT9A
PLXNA4
METAP1D
GOPC
CDON
CRELD2
CRHBP
GLOD4

Supplemental Figure 3: Degree to which Sex Modifies the Association between Inflammatory Proteome and Mortality. The mortality hazard ratio for the sex-by-biomarker interaction term (X axis) is plotted by significance level (Y axis). No proteins met statistical significance at the FDR-corrected q<0.10 level and thus are all in grey **(A)**. Pathway enrichment by gene ontology is performed on the sex-by-biomarker interaction terms for mortality to identify pathways that are more strongly associated with increased mortality among women (positive normalized enrichment score) or men (negative normalized enrichment score), with nominal p-values denoted according to the color scale **(B)**.

A. Sex-Biomarker Interaction with Mortality

Supplemental Acknowledgements

**<u>CNICS Site</u>:**	**<u>Site PI</u>**
University of Alabama Birmingham	Michael Saag/Amanda Willig
University of Washington	Mari Kitahata
University of California San Francisco	Katerina Christopoulos
University of California San Diego	Chris Mathews
Case Western Reserve University	Jeffrey Jacobson
Harvard U/Fenway Clinic	Ken Mayer
University of North Carolina Chapel Hill	Joe Eron/Sonia Napravanik
Johns Hopkins University	Richard Moore
Vanderbilt University	April Pettit
